# Crystal structure of the co-crystalline adduct 1,3,6,8-tetra­aza­tri­cyclo­[4.4.1.1^3,8^]dodecane (TATD)–4-iodo­phenol (1/2): supra­molecular assembly mediated by halogen and hydrogen bonding

**DOI:** 10.1107/S2056989017014943

**Published:** 2017-10-20

**Authors:** Augusto Rivera, Jicli José Rojas, Jaime Ríos-Motta, Michael Bolte

**Affiliations:** a Universidad Nacional de Colombia, Sede Bogotá, Facultad de Ciencias, Departamento de Química, Cra 30 No. 45-03, Bogotá, Código, Postal 111321, Colombia; b Institut für Anorganische Chemie, J. W. Goethe-Universität Frankfurt, Max-von Laue-Strasse 7, 60438 Frankfurt/Main, Germany

**Keywords:** crystal structure, co-crystalline adduct, hydrogen bonding, halogen bonding, TATD

## Abstract

The asymmetric unit of the title co-crystalline adduct, 1,3,6,8-tetra­aza­tri­cyclo[4.4.1.1^3,8^]dodecane (TATD)–4-iodo­phenol (1/2), C_8_H_16_N_4_·2C_6_H_5_IO, comprises a half mol­ecule of the aminal cage polyamine plus a 4-iodo­phenol mol­ecule. A twofold rotation axis generates the other half of the adduct. The components are linked by two inter­molecular O—H⋯N hydrogen bonds. The adducts are further linked into a three-dimensional framework structure by a combination of N⋯I halogen bonds and weak non-conventional C—H⋯O and C—H⋯I hydrogen bonds.

## Abstract 2

In the crystal of the ternary co-crystalline adduct, the components inter­act through two inter­molecular O—H⋯N hydrogen bonds. The supra­molecular adducts are inter­linked through of halogen bonds and weak non-conventional hydrogen bonds.

## Chemical context   

Halogenoorganic compounds are able to play a role in organic supra­molecular assemblies as electrophilic species, and have been used as models in the construction of self-assembled architectures. Non-covalent bonds such as hydrogen bonds (HB) and halogen bonds (XB) attract inter­est in crystal engineering because they have clear directional properties (Umezono & Okuno, 2017 ▸). Hydrogen bonds have been used successfully to construct supra­molecular architectures as a result of their high directionality, which also results in high selectivity. Halogen bonds exhibit similar directionality and strength to hydrogen bonds and can offer a new approach to the control of supra­molecular assemblies (Jin *et al.*, 2014 ▸). XB also play important roles in natural systems, and have been effectively applied in various fields including crystal engineering, solid-state mol­ecular recognition, materials with optical properties and supra­molecular liquid crystals (Li *et al.*, 2017 ▸). The strength of the inter­actions involving halogens increases on going from chlorine to bromine to iodine. Although hydrogen bonds are likely to be more effective, XB also are also important in crystal packing (Aakeröy *et al.*, 2015 ▸; Geboes *et al.*, 2017 ▸). In view of the analogies between halogen and hydrogen bonding, we think that the 4-iodo­phenol mol­ecule offers inter­esting possibilities for exploring the effect of halogen-bonding inter­actions on supra­molecular assemblies of phenols with polyamines. Following our previous work on acid–base adducts based on macrocyclic aminals and phenols, we report herein the synthesis and crystal structure of the title compound, a supra­molecular complex assembled through non-covalent HB and XB inter­actions between 4-iodo­phenol and 1,3,6,8-tetra­aza­tri­cyclo­[4.4.1.13,8]dodecane (TATD).
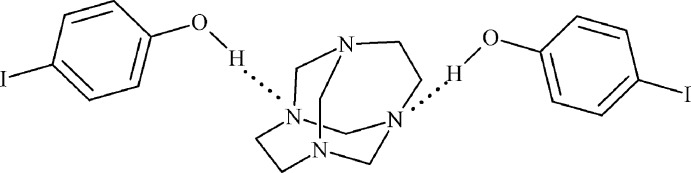


## Structural commentary   

**Figure 1:**
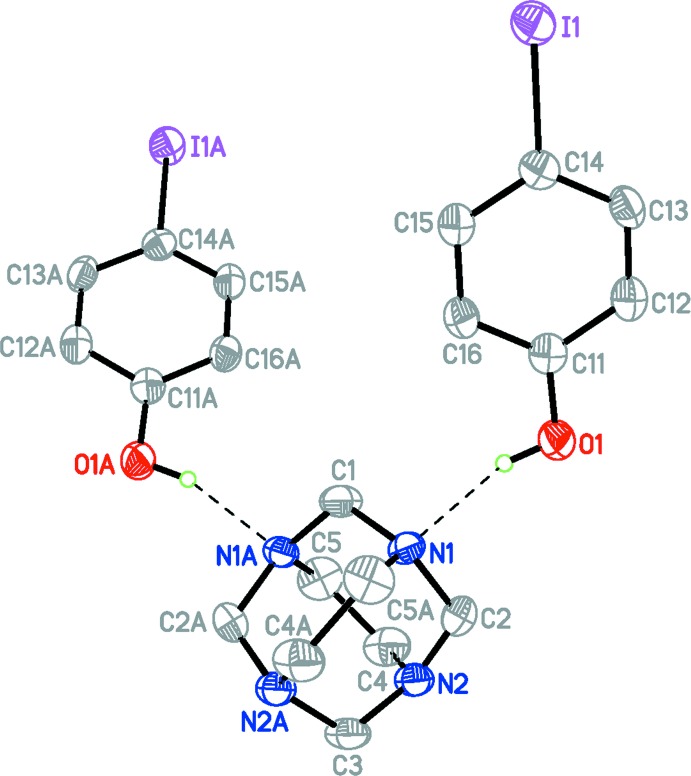
A view of the mol­ecular structure of the title compound, showing the atom-labelling scheme, with displacement ellipsoids drawn at the 50% probability. H atoms bonded to C atoms are omitted for clarity. Hydrogen bonds are drawn as dashed lines. Atoms labelled with the suffix A are generated using the symmetry operator (−*x* + 1, −*y* + 1, *z*).

The title compound is isostructural with 1,3,6,8-tetra­aza­tri­cyclo­[4.4.1.1^3,8^]dodecane (TATD)–4-bromo­phenol (Rivera, Uribe *et al.*, 2015 ▸): both crystallize in the space group *Fdd*2, and the differences between the unit-cell parameters (*a*, *b*, *c*) are < 7%. The asymmetric unit comprises one half of a 1,3,6,8-tetra­aza­tri­cyclo­[4.4.1.1^3,8^]dodecane (TATD) mol­ecule and one iodo­phenol mol­ecule held together by inter­molecular O—H⋯N hydrogen bonds [O⋯N 2.741 (6) Å; O—H⋯N 154 (7)°; Table 1 ▸]. The complete adduct is generated by a crystallographic twofold rotation axis, (Fig. 1 ▸).

Apart from the C—I/Br bond-length differences and some of the bond angles in the benzene ring, the mol­ecules have similar geometric data (bond lengths and angles). The C14—I1 bond length [2.106 (5) Å] is in good agreement with the value reported for 4-iodo­phenol itself [2.104 (5) Å; Merz, 2006 ▸]. The overall mol­ecular conformation of TATD observed here is very close to that of TATD in the related bromo­phenol adduct (Rivera, Uribe *et al.*, 2015 ▸).

## Supra­molecular features   

**Figure 2:**
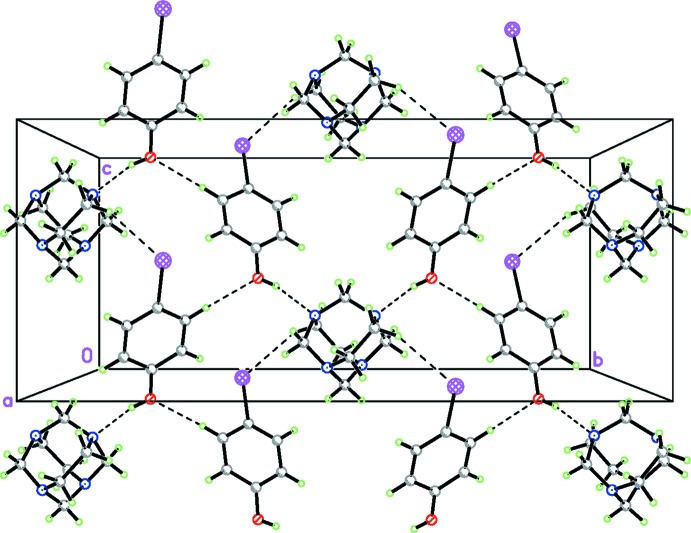
A view of the crystal packing of the title compound, showing the O—H⋯N hydrogen bonds; and C—H⋯O and C—H⋯hydrogen bonds (dashed lines).

In the crystal, the three independent mol­ecules are linked *via* two inter­molecular O1—H1⋯N1 hydrogen bonds (Table 1 ▸ and Fig. 1 ▸). These supra­molecular units are then linked by direction-specific inter­molecular inter­actions, including both non-conventional hydrogen bonds and halogen bonds, C—H⋯O and C—H⋯I hydrogen bonds, forming slabs lying parallel to the *bc* plane (Table 1 ▸ and Fig. 2 ▸). However, considering the donor–acceptor bond lengths of 3.961 (7) Å [C5—H5*B*⋯I1] and 3.455 (6) Å [C13—H13⋯O1], which exceed the sum of the corresponding van der Waals radii (0.281 and 0.255 Å, respectively), the strength of the these non-conventional hydrogen bonds can be classified as very weak (Steiner, 2003 ▸).

In addition, as indicated by a *PLATON* analysis (Spek, 2009 ▸), the iodine atom is involved, as an electron-density acceptor, in two short contacts with N2 and C3, seemingly forming a bifurcated halogen bond, where the I⋯N [3.351 (5) Å] and I⋯C distances [3.519 (5) Å] are 0.18 and 0.16 Å, respectively, less than the sum of the corresponding van der Waals radii (Alvarez, 2013 ▸). The I⋯N distance corresponds to 90% of the sum of the van der Waals radii (3.70 Å) and the C14—I1⋯N2^iii^ angle of 173.11 (2)° is close to being linear [symmetry code: (iii) *x* + 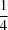, −*y* + 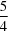, *z* + 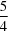]. Taking into account these geometrical parameters, the I1⋯N2 contacts can formally be considered as halogen bonds. It appears that this contact imposes the relatively close, but significantly longer I⋯C contact. Unsurprisingly, this pattern is repeated with the isostructural bromo analogue (Rivera, Uribe *et al.*, 2015 ▸) with Br⋯N = 3.292 (4) and C⋯Br = 3.477 (4) Å. There is also a Cl⋯N halogen bond in the related 4-chloro-3,5-di­methyl­phenol analogue (Rivera, Rojas, *et al.*, 2015 ▸) with Cl⋯N = 3.1680 (16); the C⋯Cl contact has extended to 3.5828 (19) Å and can be disregarded.

## Database survey   

The structure of 1,3,6,8-tetra­aza­tri­cyclo­[4.4.1.13,8]dodecane has already been determined (Murray-Rust, 1974 ▸; Rivera *et al.*, 2014 ▸). Since the mol­ecule is rigid, it is not surprising that it compares very closely with the TATD mol­ecule in the title compound. The structure of 1,3,6,8-tetra­aza­tri­cyclo[4.4.1.13,8]dodecane hydro­quinone (Rivera *et al.*, 2007 ▸) shows two O—H⋯N hydrogen bonds of similar geometry to that of the title compound. Inter­estingly, this pattern is repeated with 4-bromo­phenol 1,3,6,8-tetra­aza­tri­cyclo­[4.4.1.13,8]dodecane (Rivera, Uribe *et al.*, 2015 ▸), which is isostructural with the title compound. In contrast, 4-chloro-3,5-di­methyl­phenol 1,3,6,8-tetra­aza­tri­cyclo­[4.4.1.13,8]dodecane (Rivera, Rojas *et al.*, 2015 ▸) only forms one O—H⋯N hydrogen bond, nonetheless with similar geometric parameters to those in the title compound. Similarly, in the supra­molecular complex with a 2:1 ratio of 4-iodo­phenol to the aza-donor 1,4-di­aza­bicyclo[2.2.2]octane (Nayak & Pedireddi, 2017 ▸), the mol­ecules are again connected through O—H⋯N hydrogen bonds but with no halogen-bond inter­action involving the iodo substituent.

## Synthesis and crystallization   

A mixture of 1,3,6,8-tetra­aza­tri­cyclo­[4.4.1.1^3,8^]dodecane (TATD) (0.168g, 1 mmol) and 4-iodo­phenol (0.440g, 2 mmol) was ground at room temperature with a pestle in a mortar for 15 min., as required to complete the reaction (TLC). The mixture was recrystallized from a mixture of *n*-hexane with a few drops of ethanol to obtain crystals suitable for X-ray analysis, m.p. = 391 K. (yield: 56%).

## Refinement   

Crystal data, data collection and structure refinement details are summarized in Table 2 ▸. All H atoms were located in a difference electron-density map. The hydroxyl H atom was refined freely, while C-bound H atoms were fixed geometrically (C—H = 0.95 or 0.99 Å) and refined using a riding-model approximation, with *U*_iso_(H) set to 1.2*U*_eq_ of the parent atom

## Supplementary Material

Crystal structure: contains datablock(s) I. DOI: 10.1107/S2056989017014943/sj5537sup1.cif

Structure factors: contains datablock(s) I. DOI: 10.1107/S2056989017014943/sj5537Isup2.hkl

Supporting information file. DOI: 10.1107/S2056989017014943/sj5537Isup3.cml

CCDC reference: 1580038

Additional supporting information:  crystallographic information; 3D view; checkCIF report

## Figures and Tables

**Table 1:** Hydrogen-bond geometry (Å, °)

*D*—H⋯*A*	*D*—H	H⋯*A*	*D*⋯*A*	*D*—H⋯*A*
O1—H1⋯N1	0.84 (1)	1.96 (4)	2.741 (6)	154 (7)
C5—H5*B*⋯I1^i^	0.99	3.03	3.961 (7)	158
C13—H13⋯O1^ii^	0.95	2.53	3.455 (6)	165

Symmetry codes: (i) 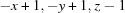; (ii) 
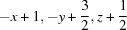
.

**Table 2:** Experimental details

Crystal data	
Chemical formula	C_8_H_16_N_4_·2C_6_H_5_IO
*M*_r_	608.25
Crystal system, space group	Orthorhombic, *F* *d* *d*2
Temperature (K)	173
*a*, *b*, *c* (Å)	20.8869 (16), 22.4197 (13), 9.6352 (6)
*V* (Å^3^)	4512.0 (5)
*Z*	8
Radiation type	Mo *K*α
μ (mm^−1^)	2.81
Crystal size (mm)	0.24 × 0.23 × 0.23

Data collection	
Diffractometer	Stoe IPDS II two-circle
Absorption correction	Multi-scan (*X-AREA*; Stoe & Cie, 2001 ▸)
*T*_min_, *T*_max_	0.548, 1.000
No. of measured, independent and observed [*I* > 2σ(*I*)] reflections	7213, 2102, 2079
*R*_int_	0.029
(sin θ/λ)_max_ (Å^−1^)	0.606

Refinement	
*R*[*F* ^2^ > 2σ(*F* ^2^)], *wR*(*F* ^2^), *S*	0.025, 0.065, 1.06
No. of reflections	2102
No. of parameters	132
No. of restraints	2
H-atom treatment	H atoms treated by a mixture of independent and constrained refinement
Δρ_max_, Δρ_min_ (e Å^−3^)	0.25, −0.67
Absolute structure	Classical Flack (1983 ▸) method preferred over Parsons because s.u. lower
Absolute structure parameter	−0.03 (4)

Computer programs: *X-AREA* (Stoe & Cie, 2001 ▸), *XP* in *SHELXTL-Plus* and *SHELXS2016* (Sheldrick, 2008 ▸) and *SHELXL2016* (Sheldrick, 2015 ▸).

## supplementary crystallographic information

### Crystal data

**Table:**

C_8_H_16_N_4_·2C_6_H_5_IO	*D*_x_ = 1.791 Mg m^−^^3^
*M**_r_* = 608.25	Mo *K*α radiation, λ = 0.71073 Å
Orthorhombic, *F**d**d*2	Cell parameters from 15571 reflections
*a* = 20.8869 (16) Å	θ = 3.6–25.9°
*b* = 22.4197 (13) Å	µ = 2.81 mm^−^^1^
*c* = 9.6352 (6) Å	*T* = 173 K
*V* = 4512.0 (5) Å^3^	Block, yellow
*Z* = 8	0.24 × 0.23 × 0.23 mm
*F*(000) = 2368	

### Data collection

**Table:**

Stoe IPDS II two-circle diffractometer	2079 reflections with *I* > 2σ(*I*)
Radiation source: Genix 3D IµS microfocus X-ray source	*R*_int_ = 0.029
ω scans	θ_max_ = 25.5°, θ_min_ = 3.6°
Absorption correction: multi-scan (X-AREA; Stoe & Cie, 2001)	*h* = −24→25
*T*_min_ = 0.548, *T*_max_ = 1.000	*k* = −26→27
7213 measured reflections	*l* = −11→11
2102 independent reflections	

### Refinement

**Table:**

Refinement on *F*^2^	Hydrogen site location: mixed
Least-squares matrix: full	H atoms treated by a mixture of independent and constrained refinement
*R*[*F*^2^ > 2σ(*F*^2^)] = 0.025	*w* = 1/[σ^2^(*F*_o_^2^) + (0.0495*P*)^2^ + 5.1297*P*] where *P* = (*F*_o_^2^ + 2*F*_c_^2^)/3
*wR*(*F*^2^) = 0.065	(Δ/σ)_max_ = 0.002
*S* = 1.06	Δρ_max_ = 0.25 e Å^−^^3^
2102 reflections	Δρ_min_ = −0.67 e Å^−^^3^
132 parameters	Absolute structure: Classical Flack (1983) method preferred over Parsons because s.u. lower
2 restraints	Absolute structure parameter: −0.03 (4)

### Special details

**Table:**

Geometry. All esds (except the esd in the dihedral angle between two l.s. planes) are estimated using the full covariance matrix. The cell esds are taken into account individually in the estimation of esds in distances, angles and torsion angles; correlations between esds in cell parameters are only used when they are defined by crystal symmetry. An approximate (isotropic) treatment of cell esds is used for estimating esds involving l.s. planes.

### Fractional atomic coordinates and isotropic or equivalent isotropic displacement parameters (Å^2^)

**Table:**

	*x*	*y*	*z*	*U*_iso_*/*U*_eq_	Occ. (<1)
N1	0.5189 (2)	0.55327 (17)	0.2785 (4)	0.0293 (9)	
N2	0.4501 (2)	0.5309 (2)	0.0695 (5)	0.0335 (9)	
C1	0.500000	0.500000	0.3551 (8)	0.0350 (16)	
H1A	0.463825	0.511142	0.416230	0.042*	0.5
H1B	0.536176	0.488858	0.416229	0.042*	0.5
C2	0.4734 (3)	0.5730 (3)	0.1705 (6)	0.0413 (13)	
H2A	0.435585	0.589927	0.218543	0.050*	
H2B	0.493847	0.606119	0.119081	0.050*	
C3	0.500000	0.500000	−0.0073 (9)	0.0383 (16)	
H3A	0.521109	0.529565	−0.068331	0.046*	0.5
H3B	0.478891	0.470437	−0.068336	0.046*	0.5
C4	0.3977 (3)	0.4922 (3)	0.1176 (7)	0.0439 (13)	
H4A	0.381691	0.469170	0.037109	0.053*	
H4B	0.362214	0.517973	0.149924	0.053*	
C5	0.4138 (3)	0.4485 (3)	0.2331 (7)	0.0456 (14)	
H5A	0.386853	0.458252	0.314539	0.055*	
H5B	0.401546	0.407971	0.201822	0.055*	
I1	0.63999 (2)	0.68958 (2)	1.00445 (6)	0.03902 (14)	
O1	0.5030 (2)	0.65445 (17)	0.4344 (4)	0.0364 (8)	
H1	0.518 (3)	0.623 (2)	0.402 (7)	0.05 (2)*	
C11	0.5320 (2)	0.6593 (2)	0.5604 (6)	0.0293 (10)	
C12	0.5220 (3)	0.7116 (2)	0.6361 (6)	0.0352 (11)	
H12	0.494289	0.741464	0.600328	0.042*	
C13	0.5518 (2)	0.72031 (19)	0.7624 (7)	0.0330 (10)	
H13	0.544963	0.756109	0.813067	0.040*	
C14	0.5920 (2)	0.6759 (2)	0.8149 (6)	0.0293 (10)	
C15	0.6015 (2)	0.6234 (2)	0.7428 (7)	0.0312 (9)	
H15	0.628314	0.593158	0.780345	0.037*	
C16	0.5715 (2)	0.6147 (2)	0.6143 (6)	0.0309 (10)	
H16	0.578032	0.578773	0.564169	0.037*	

### Atomic displacement parameters (Å^2^)

**Table:**

	*U*^11^	*U*^22^	*U*^33^	*U*^12^	*U*^13^	*U*^23^
N1	0.038 (2)	0.028 (2)	0.022 (2)	−0.0009 (16)	−0.0024 (17)	0.0024 (16)
N2	0.031 (2)	0.045 (2)	0.025 (2)	0.0090 (19)	0.0002 (17)	0.0008 (18)
C1	0.047 (4)	0.035 (4)	0.023 (3)	−0.008 (3)	0.000	0.000
C2	0.050 (3)	0.038 (3)	0.036 (3)	0.015 (3)	−0.006 (2)	0.004 (2)
C3	0.032 (3)	0.061 (4)	0.022 (3)	0.011 (3)	0.000	0.000
C4	0.026 (2)	0.067 (4)	0.038 (3)	0.007 (2)	−0.003 (2)	0.002 (3)
C5	0.039 (3)	0.050 (3)	0.048 (4)	−0.007 (2)	−0.001 (3)	0.002 (3)
I1	0.0425 (2)	0.03403 (19)	0.0405 (2)	−0.00169 (13)	−0.00853 (15)	−0.00435 (16)
O1	0.044 (2)	0.0297 (18)	0.0355 (19)	0.0035 (16)	−0.0077 (17)	−0.0043 (16)
C11	0.024 (2)	0.030 (2)	0.034 (2)	−0.0028 (18)	0.0035 (19)	0.0018 (19)
C12	0.035 (3)	0.029 (2)	0.041 (3)	0.005 (2)	0.001 (2)	0.000 (2)
C13	0.037 (2)	0.0255 (19)	0.036 (3)	0.0028 (18)	0.007 (2)	−0.005 (3)
C14	0.027 (2)	0.032 (2)	0.030 (2)	−0.0029 (19)	0.003 (2)	0.000 (2)
C15	0.030 (2)	0.027 (2)	0.036 (3)	0.0023 (16)	0.001 (2)	0.001 (2)
C16	0.033 (2)	0.024 (2)	0.036 (2)	−0.0012 (18)	0.002 (2)	−0.002 (2)

### Geometric parameters (Å, º)

**Table:**

N1—C1	1.458 (5)	C5—H5A	0.9900
N1—C5^i^	1.474 (7)	C5—H5B	0.9900
N1—C2	1.478 (7)	I1—C14	2.106 (5)
N2—C2	1.440 (8)	O1—C11	1.361 (6)
N2—C3	1.453 (6)	O1—H1	0.838 (14)
N2—C4	1.472 (8)	C11—C16	1.395 (7)
C1—H1A	0.9900	C11—C12	1.397 (8)
C1—H1B	0.9900	C12—C13	1.381 (9)
C2—H2A	0.9900	C12—H12	0.9500
C2—H2B	0.9900	C13—C14	1.398 (8)
C3—H3A	0.9900	C13—H13	0.9500
C3—H3B	0.9900	C14—C15	1.382 (7)
C4—C5	1.520 (9)	C15—C16	1.401 (8)
C4—H4A	0.9900	C15—H15	0.9500
C4—H4B	0.9900	C16—H16	0.9500
			
C1—N1—C5^i^	112.8 (4)	C5—C4—H4B	108.2
C1—N1—C2	115.3 (4)	H4A—C4—H4B	107.3
C5^i^—N1—C2	114.4 (5)	N1^i^—C5—C4	116.4 (5)
C2—N2—C3	114.5 (4)	N1^i^—C5—H5A	108.2
C2—N2—C4	115.1 (5)	C4—C5—H5A	108.2
C3—N2—C4	114.4 (4)	N1^i^—C5—H5B	108.2
N1—C1—N1^i^	119.2 (6)	C4—C5—H5B	108.2
N1—C1—H1A	107.5	H5A—C5—H5B	107.3
N1^i^—C1—H1A	107.5	C11—O1—H1	103 (5)
N1—C1—H1B	107.5	O1—C11—C16	122.6 (5)
N1^i^—C1—H1B	107.5	O1—C11—C12	117.7 (5)
H1A—C1—H1B	107.0	C16—C11—C12	119.6 (5)
N2—C2—N1	119.8 (4)	C13—C12—C11	120.8 (5)
N2—C2—H2A	107.4	C13—C12—H12	119.6
N1—C2—H2A	107.4	C11—C12—H12	119.6
N2—C2—H2B	107.4	C12—C13—C14	119.3 (5)
N1—C2—H2B	107.4	C12—C13—H13	120.4
H2A—C2—H2B	106.9	C14—C13—H13	120.4
N2—C3—N2^i^	118.8 (7)	C15—C14—C13	120.8 (5)
N2—C3—H3A	107.6	C15—C14—I1	119.5 (4)
N2^i^—C3—H3A	107.6	C13—C14—I1	119.8 (4)
N2—C3—H3B	107.6	C14—C15—C16	119.8 (5)
N2^i^—C3—H3B	107.6	C14—C15—H15	120.1
H3A—C3—H3B	107.0	C16—C15—H15	120.1
N2—C4—C5	116.5 (5)	C11—C16—C15	119.7 (5)
N2—C4—H4A	108.2	C11—C16—H16	120.2
C5—C4—H4A	108.2	C15—C16—H16	120.2
N2—C4—H4B	108.2		
			
C5^i^—N1—C1—N1^i^	−82.7 (4)	O1—C11—C12—C13	−177.7 (5)
C2—N1—C1—N1^i^	51.3 (3)	C16—C11—C12—C13	1.5 (8)
C3—N2—C2—N1	−55.1 (7)	C11—C12—C13—C14	−0.5 (8)
C4—N2—C2—N1	80.5 (7)	C12—C13—C14—C15	−0.9 (8)
C1—N1—C2—N2	−50.6 (7)	C12—C13—C14—I1	178.2 (4)
C5^i^—N1—C2—N2	82.6 (7)	C13—C14—C15—C16	1.3 (8)
C2—N2—C3—N2^i^	53.6 (4)	I1—C14—C15—C16	−177.8 (4)
C4—N2—C3—N2^i^	−82.3 (4)	O1—C11—C16—C15	178.1 (5)
C2—N2—C4—C5	−65.6 (7)	C12—C11—C16—C15	−1.1 (8)
C3—N2—C4—C5	70.1 (7)	C14—C15—C16—C11	−0.3 (8)
N2—C4—C5—N1^i^	−3.8 (8)		

Symmetry code: (i) −*x*+1, −*y*+1, *z*.

### Hydrogen-bond geometry (Å, º)

**Table:**

*D*—H···*A*	*D*—H	H···*A*	*D*···*A*	*D*—H···*A*
O1—H1···N1	0.84 (1)	1.96 (4)	2.741 (6)	154 (7)
C5—H5*B*···I1^ii^	0.99	3.03	3.961 (7)	158
C13—H13···O1^iii^	0.95	2.53	3.455 (6)	165

Symmetry codes: (ii) −*x*+1, −*y*+1, *z*−1; (iii) −*x*+1, −*y*+3/2, *z*+1/2.
